# *Neobacillus nitrireducens* sp. nov., a soil bacterium performing nitrate reduction under low-temperature and microaerobic conditions

**DOI:** 10.3389/fmicb.2026.1842410

**Published:** 2026-05-11

**Authors:** Jinwoo Ahn, Satoshi Ishii, Tatsuya Unno, Jeonghwan Jang

**Affiliations:** 1 Division of Biotechnology and Advanced Institute of Environment and Bioscience, Jeonbuk National University, Iksan, Republic of Korea; 2 BioTechnology Institute, University of Minnesota, St. Paul, MN, United States; 3 Department of Soil, Water, and Climate, University of Minnesota, St. Paul, MN, United States; 4 Department of Biological Sciences and Biotechnology, Chungbuk National University, Cheongju, Republic of Korea

**Keywords:** aerobic nitrate reduction, cold-adapted, *Neobacillus* species novel, soil bacterium, whole genome sequence

## Abstract

A facultative anaerobic spore-forming bacterium, designated OS1-33^T^ was isolated from apple orchard soil collected at Iksan-si, Republic of Korea. The phylogenetic position of the strain OS1-33^T^ was investigated by 16S rRNA gene sequence similarity analysis showing that it tightly clustered with species of the genus *Neobacillus*. The highest sequence similarity was observed to *Neobacillus drentensis* (99.93%). The genome of OS1-33^T^ showed an Average Nucleotide Identity (ANI) of less than 95% with all the other known type strains of *Neobacillus* species including *N. drentensis* DSM 15600^T^. In addition, the digital DNA–DNA hybridization (dDDH) value between strain OS1-33^T^ and *N. drentensis* was 52.4% (95% confidence interval, 49.8%−55.1%), which is clearly below the 70% threshold for species delineation. The DNA G+C content of OS1-33^T^ was determined to be 38.92 mol%. Strain OS1-33^T^ exhibited nitrate reduction activity despite the presence of oxygen and at low temperature, suggesting that it may serve as a valuable microbial resource for nitrate removal in bioreactors operated under low-temperature conditions. Furthermore, distinct morphological, physiological, biochemical, and genomic characteristics differentiated strain OS1-33^T^ from its closest relatives. Based on overall results of the comparative analyses, strain OS1-33^T^ is proposed to represent a novel species within the genus *Neobacillus*, for which the name *Neobacillus nitrireducens* sp. nov. is proposed. The type strain is OS1-33^T^ (=KCCM 43525^T^ = JCM 38007^T^).

## Introduction

Agricultural practices aimed at maximizing crop productivity frequently involve the excessive application of nitrogen fertilizers, such as urea and animal manure, which are eventually broken down into ammonium (Park and Jang, 2024). Ammonium in soil can be oxidized to nitrite and nitrate by nitrifying organisms, resulting in a gradual increase in nitrate concentrations in soil (Dong et al., 2022; Wang and Ishii, 2025). Excess nitrate accumulated in soil can be readily leached into groundwater, rivers, and lakes during rainfall or irrigation events, leading to eutrophication and ultimately causing serious environmental problems such as water quality deterioration and loss of biodiversity (Craswell, 2021). Therefore, the biological reduction of nitrate to nitrogen gas, known as denitrification, plays a crucial role in maintaining sustainable nitrogen cycling and mitigating environmental pollution (Wang and Ishii, 2025). However, denitrification typically occurs under anaerobic conditions and is often inhibited by low temperatures or high oxygen concentrations (Stanford et al., 1975; Hochstein et al., 1984). Despite the important role of denitrification in soil nitrogen cycling, relatively few studies have investigated denitrifying microorganisms capable of maintaining stable activity under such suboptimal conditions. The isolation of microorganisms exhibiting efficient denitrification under these conditions is therefore essential for developing effective bioremediation strategies to address nitrate contamination in agricultural environments.

The genus *Neobacillus* was proposed by Patel and Gupta (Patel and Gupta, 2020) based on the analyses of conserved signature indels and whole-genome phylogenetic data. This genus comprises several species that were previously classified within the genus *Bacillus* (Patel and Gupta, 2020). According to the List of Prokaryotic Names with Standing in Nomenclature (https://lpsn.dsmz.de/genus/neobacillus), the genus *Neobacillus* currently includes 27 validly published species. Members of this genus have been isolated from a wide range of environments, including soil (Nagel and Andreesen, 1991; Heyrman et al., 2004; Ten et al., 2007; Nguyen et al., 2013; Yuki et al., 2022; Han et al., 2023), windrow compost (Han et al., 2013), deep-sea sediments (Yu et al., 2019), fumarole-associated habitats (Patel and Gupta, 2020), plant roots (Liu et al., 2014; Kämpfer et al., 2016; Zhang et al., 2017; Jiang et al., 2021), and human feces (Bittar et al., 2015). In recent years, studies on novel denitrifying microorganisms have increased, and several strains belonging to the genus *Neobacillus* have also been reported to exhibit denitrification capability (Ahn and Jang, 2025; Ahn et al., 2025). However, systematic investigations of their denitrification potential and ecological roles remain limited.

In this study, we isolated and characterized a bacterial strain, OS1-33^T^, from apple orchard soil and investigated its taxonomic position using a polyphasic taxonomic approach, including genomic, morphological, physiological, and chemotaxonomic analyses. Comparative analyses with phylogenetically related species revealed that strain OS1-33^T^ represents a distinct phylogenetic lineage within the genus *Neobacillus*. In addition, the denitrification capability of strain OS1-33^T^ was evaluated under conditions different from those typically associated with conventional denitrifiers, including low temperature with presence of oxygen. The efficient nitrate removal observed under these atypical conditions suggests that strain OS1-33^T^ may represent a valuable microbial resource for the development of biological treatment technologies aimed at mitigating nitrate pollution in environmental systems.

Based on the results of this study, strain OS1-33^T^ exhibits characteristics that clearly distinguish it from previously described species within the genus *Neobacillus*. Therefore, we propose that this strain represents a novel species of the genus *Neobacillus*, for which the name *Neobacillus nitrireducens* sp. nov. is proposed.

## Materials and methods

### Bacterial strains

Strain OS1-33^T^ was isolated from surface soil collected at a depth of 0–2 cm from an apple orchard in Iksan-si, Jeollabuk-do, Republic of Korea (35°58′44.03″N, 127°03′31.33″E). To obtain the strain, 2 g of soil was suspended in 10 ml of sterile phosphate-buffered saline (PBS, pH 7.0). After a 1:100 dilution, 100 μl of the suspension was spread onto R2A agar plates (MB Cell, Republic of Korea). The plates were incubated anaerobically at 28°C for 5 days using a GasPak EZ Anaerobe system (BD, USA). Resulting colonies were purified by repeated streaking onto fresh R2A agar plates to obtain single, well-isolated colonies. One purified colony was designated as strain OS1-33^T^ based on its confirmed nitrate (${\text{NO}}_{3}^{-}$) removal activity determined using a colorimetric assay (Heo et al., 2020).

A pure culture of *Neobacillus drentensis* DSM 15600^T^, the type strain of the phylogenetically closest species, was obtained from the Deutsche Sammlung von Mikroorganismen und Zellkulturen (DSMZ, Germany) for comparative analysis of phenotypic characteristics and nitrate removal efficiency with strain OS1-33^T^.

### Screening of strains with nitrate (${\text{NO}}_{3}^{-}$) removal activity

To evaluate the nitrate (${\text{NO}}_{3}^{-}$) removal capability of soil isolates, pure single colonies were inoculated into individual wells of a 96-well-cell culture microplate (SPL Life Sciences, Republic of Korea) containing 200 μl of R2A broth (MB Cell, Republic of Korea) supplemented with 5 mM KNO3 and 10 mM acetate. The cultures were incubated at 28°C for 5 days under aerobic and anaerobic conditions, with anaerobic incubation performed using a GasPak EZ Anaerobe system (BD, USA).

The nitrate concentration in the culture supernatant was determined using a modified colorimetric microplate assay based on previously described methods (Heo et al., 2020). Briefly, 5 μl of culture supernatant was transferred to a new 96-well-plate and diluted 20-fold by adding 95 μl of sterile distilled water. Subsequently, 80 μl of modified Griess reagent (Sigma-Aldrich, USA) was added to each well. After 30 min, absorbance of each well was measured at 540 nm using a Multiskan SkyHigh Microplate Spectrophotometer (Thermo Fisher Scientific, USA). After that, 20 μl of vanadium(III) chloride solution (1% w/v in 1 M HCl) was added and then incubated at 60°C for 1 h, followed by another absorbance measurement at 540 nm. Nitrate concentrations were calculated using standard solutions ranging from 0.01 to 0.5 mM.

### Genomic analyses

Genomic DNA of strain OS1-33^T^ and *N. drentensis* DSM 15600^T^ was extracted using the DNeasy PowerLyzer Microbial Kit (Qiagen, USA) according to the manufacturer's instructions. The extracted DNA was eluted in 50 μl of buffer, and its quality and concentration were evaluated using a Multiskan SkyHigh Microplate Spectrophotometer (Thermo Fisher Scientific, USA) equipped with a μDrop Duo Plate. The DNA showed A260/280 and A260/230 ratios of 1.8–2.0 and 2.0–2.2, respectively, with a concentration exceeding 4,000 ng.

The 16S rRNA gene of strain OS1-33^T^ was amplified using the universal primers 27F (5′-AGA GTT TGA TCM TGG CTC AG-3′) and 1492R (5′-CGG TTA CCT TGT TAC GAC TT-3′) as previously described (Frank et al., 2008). The amplified PCR products were sequenced using an ABI 3730*xl* DNA Analyzer (Thermo Fisher Scientific, USA) by SolGent Co., Ltd. (Daejeon, Republic of Korea). The sequence reads were assembled using Phred/Phrap/Consed assembler version 29 (http://www.phrap.com). The 16S rRNA gene sequence of strain OS1-33^T^ was deposited in the NCBI GenBank database under accession number PV185345. Phylogenetic analysis based on 16S rRNA gene sequences was performed using the maximum-likelihood method and neighbor-joining method implemented in MEGA X software (Kumar et al., 2018), and the robustness of the tree topology was evaluated using bootstrap analysis with 1,000 replicates.

Long-read whole-genome sequencing of strain OS1-33^T^ and DSM 15600^T^ was performed using the Oxford Nanopore MinION platform (Oxford Nanopore Technologies, UK) at the NGS Center of Kyungpook National University (Daegu, Republic of Korea). Sequencing libraries were prepared using the Native Barcoding Kit 24 V14 and sequenced on an R10.4.1 flow cell with a MinION Mk1c system. Base calling was performed using Guppy Basecaller v2.24 (Wick et al., 2019). Reads shorter than 1 kb or within the lowest 5% quality scores were removed using Filtlong v0.2.1. *De novo* genome assemblies was conducted using Flye v2.9.1 (Kolmogorov et al., 2019), and the assembled genomes were deposited in GenBank under accession number NZ_CP133262 and CM147946 for strain OS1-33^T^ and DSM 15600^T^, respectively. Functional annotation and gene prediction were performed using the NCBI Prokaryotic Genome Annotation Pipeline (PGAP; Supplementary Table S1; Tatusova et al., 2016).

To determine the taxonomic position of strain OS1-33^T^, average nucleotide identity (ANI) values were calculated between strain OS1-33^T^ and other members of the genus *Neobacillus* using the OrthoANIu algorithm (Yoon et al., 2017). Genome sequences of the closely related *Neobacillus* type strains were obtained from the NCBI database except for the one of strain DSM 15600^T^. The percentage of conserved proteins (POCP) among *Neobacillus* genomes was calculated following the method described by Qin et al. (2014). The genomic G+C contents were calculated from the whole-genome sequence using the G+C Content Calculator available at EZBioCloud (https://www.ezbiocloud.net/tools/ani). Additionally, to further evaluate the taxonomic position of strain OS1-33^T^, digital DNA–DNA hybridization (dDDH) values against type strains were calculated using the GGDC platform (https://ggdc.dsmz.de/distcalc2.php; Auch et al., 2010), and a genome-based phylogenomic tree was constructed using the Type (Strain) Genome Server (TYGS). The tree was inferred using the Genome BLAST Distance Phylogeny (GBDP) approach implemented in the Type Strain Genome Server (Meier-Kolthoff and Göker, 2019; Meier-Kolthoff et al., 2022; Freese et al., 2026). Pairwise intergenomic distances were calculated using the GBDP method under the “trimming” algorithm and distance formula d5 (Meier-Kolthoff et al., 2013). The tree was constructed using FastME with 100 pseudo-bootstrap replicates, and branch support values (%) >60% are shown at the nodes (Lefort et al., 2015; Farris, 1972). Branch lengths represent GBDP distance values.

### Morphological and physiological analyses

Colony characteristics of strain OS1-33^T^ and *N. drentensis* DSM 15600^T^ were examined after aerobic incubation on R2A agar (MB Cell, Republic of Korea) at 30°C for 2 days. Gram staining was performed using the Gram Color Kit (MB Cell, Republic of Korea), and spore staining was carried out using the Spore Color Kit (MB Cell, Republic of Korea) according to the manufacturer's instructions (Schaeffer and Fulton, 1933).

Catalase activity was determined by adding a small amount of bacterial culture to 3 ml of 3% hydrogen peroxide (H_2_O_2_) in a test tube and observing the formation of oxygen bubbles.

Growth was examined in R2A broth using glass test tubes (Techno Glass, Japan) under various temperatures (10°C, 15°C, 20°C, 25°C, 30°C, 35°C, and 40°C), pH conditions (pH 5, 6, 7, 8, 9, 10, 11, and 12), and NaCl concentrations (0%, 0.5%, 1%, 2%, 3%, 4%, and 5%, w/v) during a 3 day incubation with shaking at 100 rpm on an orbital shaker. In addition, growth of strain OS1-33^T^ at 10°C was observed after 2 weeks of incubation.

Carbon source utilization was tested using the API 50CH kit (bioMérieux, France). Cellular fatty acid composition and respiratory quinones were analyzed by the Korean Culture Center of Microorganisms (Seoul, Republic of Korea) according to the MIDI protocol (Sasser, 2006).

The cellular structure of strain OS1-33^T^ was examined using a Hitachi H-7650 transmission electron microscope (Hitachi, Japan) at the Center for University-Wide Research Facilities, Jeonbuk National University (Jeonju, Republic of Korea).

### Nitrate (${\text{NO}}_{3}^{-}$) removal under various environmental conditions

To evaluate nitrate removal under low-temperature condition, concentrated cells of strain OS1-33^T^ and *N. drentensis* DSM 15600^T^ suspended in PBS were inoculated into R2A broth supplemented with 10 mM acetate and 5 mM KNO3 in glass test tubes (Techno Glass, Japan) and Hungate anaerobic culture tubes (Chemglass, USA), with the initial OD600 of the cultures adjusted to 2.2. The Hungate tubes were flushed with nitrogen gas (N_2_), sealed, and incubated at 10°C for 7 days.

To evaluate nitrate removal under different oxygen conditions, R2A broth supplemented with 10 mM acetate and 5 mM KNO^3^ in glass test tubes (Techno Glass, Japan) inoculated with a single colony of strain OS1-33^T^ were incubated at 30°C for 48 h while varying the shaking speed (25–100 rpm) to control the dissolved oxygen (DO) concentration. Subsequently, the DO in the culture supernatant was measured using a portable multimeter AM70 (Apera Instruments, USA). The DO in our experiment ranged from 0.99 to 7.81 mg/L.

Under the same temperature conditions (30°C), a separate experiment was conducted to compare the nitrate removal performance of strain OS1-33^T^ and *N. drentensis* DSM 15600^T^. R2A broth supplemented with 10 mM acetate and 5 mM KNO3 in glass test tubes (Techno Glass, Japan) was inoculated with a single colony of strain OS1-33^T^ or DSM 15600^T^ and incubated at 30°C with shaking at 100 rpm for 72 h.

After incubation, bacterial growth was determined by measuring the optical density at 600 nm (OD_600_) using a GENESYS 30 Visible Spectrophotometer (Thermo Fisher Scientific, USA). The concentration of nitrate (${\text{NO}}_{3}^{-}$) in the culture supernatant was quantified using the colorimetric microplate assay described previously (Heo et al., 2020). All experiments were performed in triplicate.

## Results and discussion

### Genomic taxonomy

**Figure 1:**
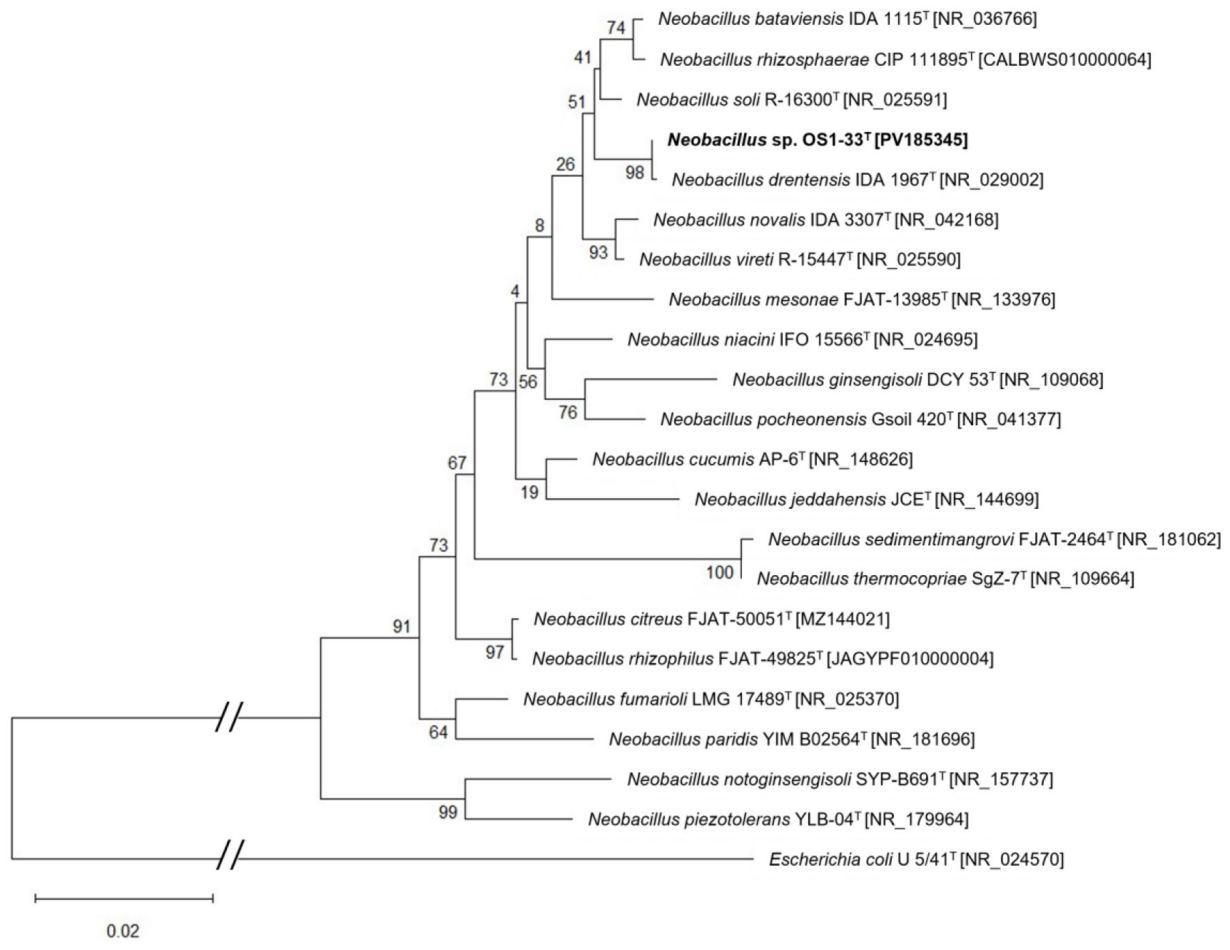
Phylogenetic relationships of *Neobacillus* species based on 16S rRNA gene sequences. The tree was constructed using the maximum-likelihood method. GenBank accession numbers are shown in square brackets. Bootstrap values (%) based on 1,000 replicates are indicated at the branch nodes. Branch lengths represent sequence divergence, as indicated by the scale bar. The tree shows that *Neobacillus drentensis* IDA 1967^T^ (=DSM 15600^T^) is most closely related to *Neobacillus nitrireducens* OS1-33^T^.

**Figure 2:**
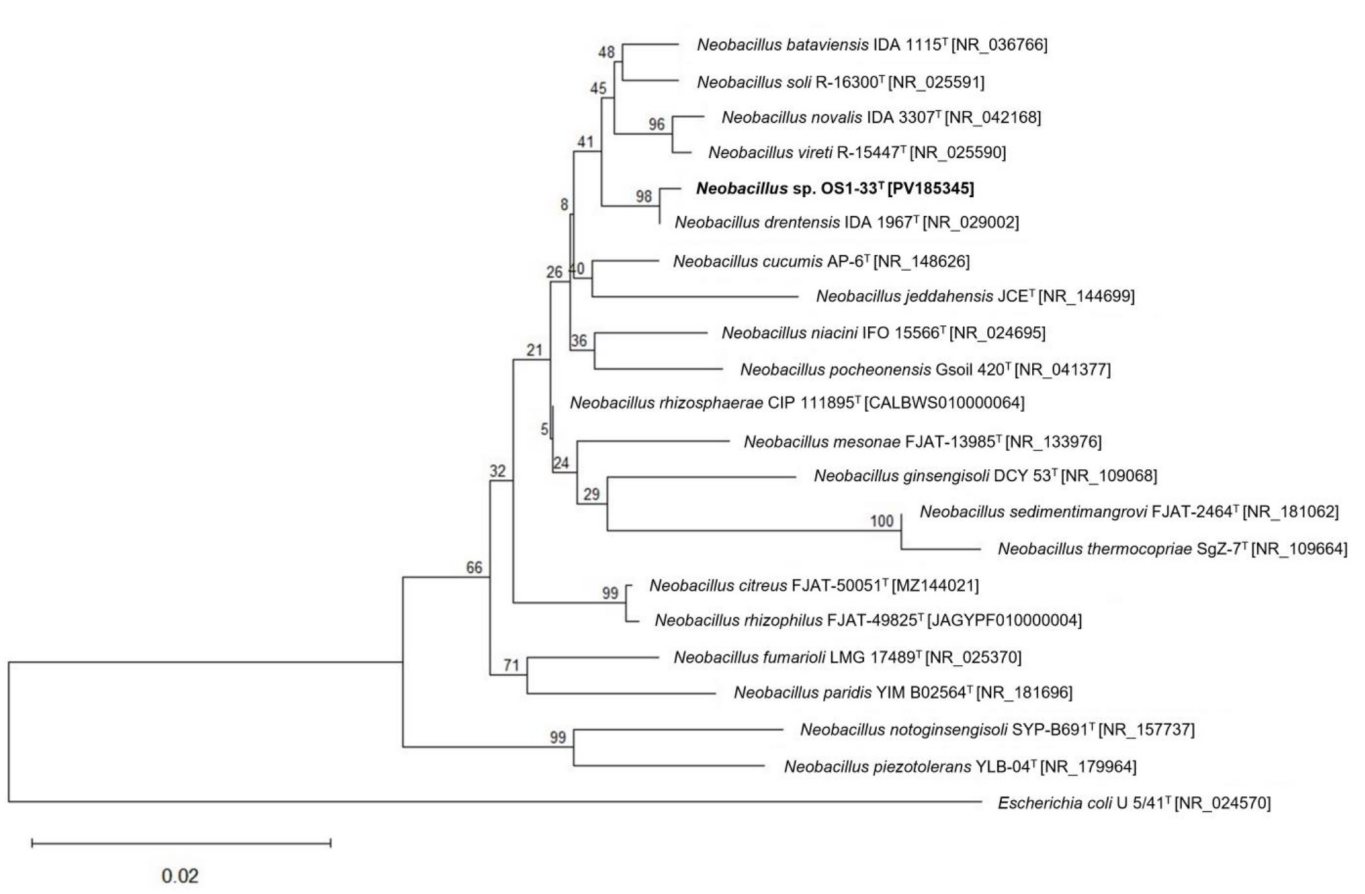
Phylogenetic relationships of *Neobacillus* species based on 16S rRNA gene sequences. The tree was constructed using the neighbor-joining method. GenBank accession numbers are shown in square brackets. Bootstrap values (%) based on 1,000 replicates are indicated at the branch nodes. Branch lengths represent sequence divergence, as indicated by the scale bar. The tree shows that *Neobacillus drentensis* IDA 1967^T^ (=DSM 15600^T^) is most closely related to *Neobacillus nitrireducens* OS1-33^T^.

The near complete 16S rRNA gene sequence (1,538 bp) of strain OS1-33^T^ was analyzed and compared with those of validly published type strains within the genus *Neobacillus*. Strain OS1-33^T^ showed the highest sequence similarity to *N. drentensis* IDA 1967^T^(=DSM 15600^T^; 99.93%). Also, strain OS1-33^T^ also showed high 16S rRNA gene sequence similarity to *N. bataviensis* NBRC 102449^T^ (=LMG 21833^T^ = IDA 1115^T^; 99.28%), followed by *N. niacini* NBRC 15566 ^T^ (=IFO 15566 ^T^; 99.06%), *N. cucumis* AP-6^T^ (=DSM 101566^T^; 98.92%), *N. novalis* NBRC 102450 ^T^ (=IDA 3307 ^T^; 98.92%), *N. rhizosphaerae* CIP 111895^T^ (98.27%), *N. soli* R-16300^T^ (DSM 15604^T^; 99.27%), *N. vireti* R-15447^T^ (=DSM 15602^T^; 99.05%), *N. mesonae* FJAT-13985^T^ (98.27%), *N. ginsengisoli* DCY 53^T^ (=DSM 27594^T^; 98.13%), and *N. pocheonensis* Gsoil420^T^ (=KCTC 13943^T^; 98.19%). Based on the phylogenetic tree generated using the maximum-likelihood method and neighbor-joining method, strain OS1-33^T^ was clustered together with *N. drentensis* IDA 1967^T^ (=DSM 15600^T^), which was supported by high bootstrap values (Figures 1, 2). However, it is well-recognized that 16S rRNA gene sequences often lack sufficient resolution for species-level discrimination in *Bacillus* and related taxa due to their high conservation, which leads to diminished reliability in taxonomic identification at the species level (Maughan and Van Der Auwera, 2011).

To further elucidate the genomic relationships among the strains, the whole-genome sequence of strain OS1-33^T^ was identified (see sequence summary in Supplementary Table S1) and compared with reference genomes retrieved from the NCBI GenBank database. The genomic DNA G+C content of strain OS1-33^T^ was 38.92%, which is similar to that of *N. drentensis* DSM 15600^T^ (38.93%) and other *Neobacillus* species (Supplementary Table S2).

**Figure 3:**
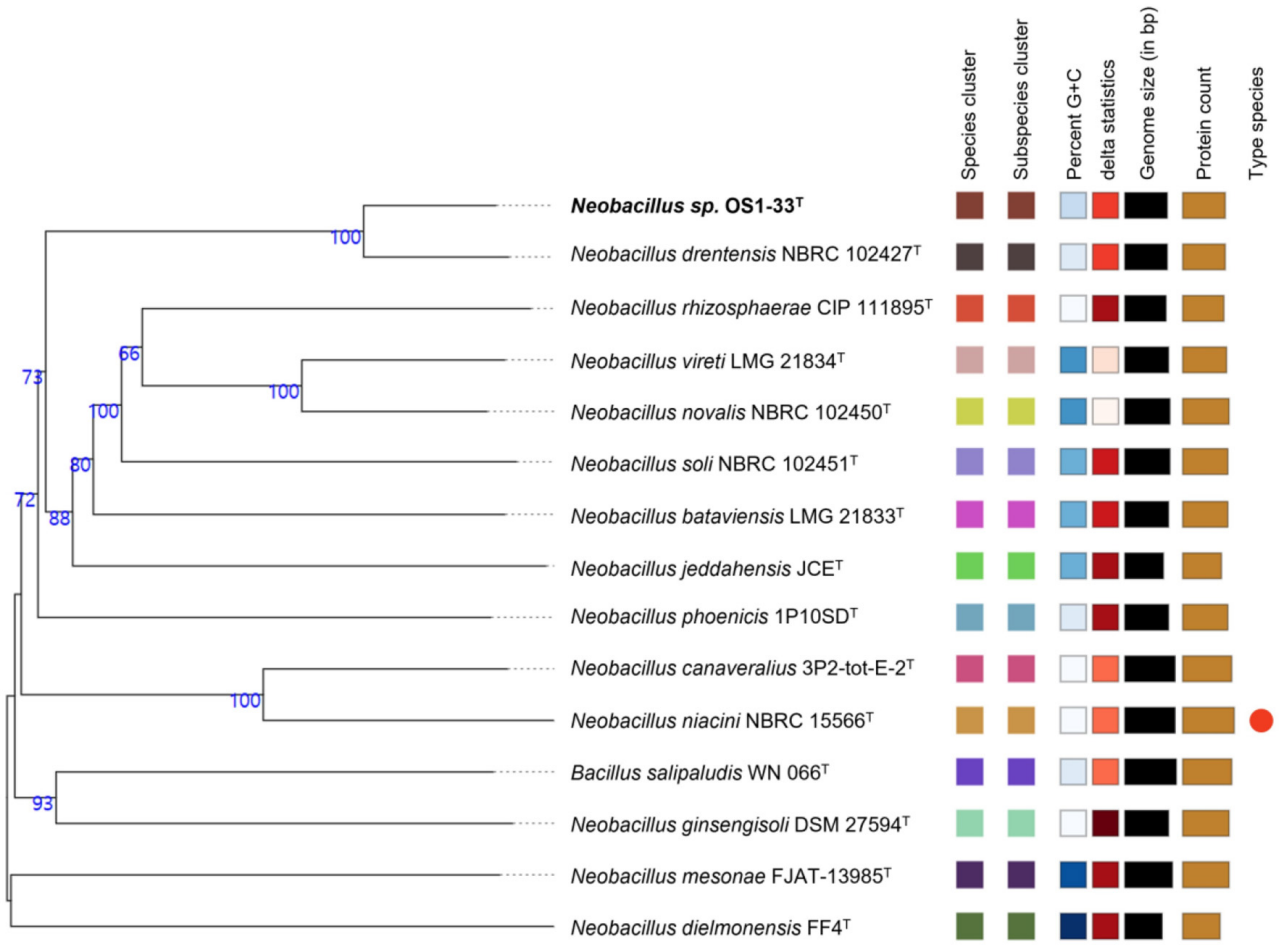
Phylogenomic relationships of *Neobacillus* species based on whole-genome sequences. The tree was inferred using the Genome BLAST Distance Phylogeny (GBDP) approach implemented in the Type Strain Genome Server.

Whole-genome-based average nucleotide identity (ANI) comparisons between strain OS1-33^T^ and the type strains of related species revealed a maximum ANI value of 93.19% with *N. drentensis* (Table 1), which is below the 95% ANI threshold generally accepted for species delineation (Richter and Rosselló-Móra, 2009). The dDDH value between strain OS1-33^T^ and *N. drentensis* was 52.4% (95% confidence interval: 49.8%−55.1%), which is clearly below the 70% threshold for species delineation (Table 1). Furthermore, the ANI and dDDH values between strain OS1-33^T^ and the other type strains were also below 95 and 70%, respectively (Table 1). TYGS genome-based phylogenomic analysis also placed strain OS1-33^T^ within the genus *Neobacillus* as a distinct lineage closely related to *N. drentensis*, further supporting its recognition as a novel species despite the very high 16S rRNA gene sequence similarity (Figure 3). These genomic results support the classification of strain OS1-33^T^ as a novel species within the genus *Neobacillus*, closely related to *N. drentensis*.

**Table 1:** Average nucleotide identity (ANI), digital DNA-DNA hybridization (dDDH), and percentage of conserved proteins (POCP) values between strain OS1-33^T^ and type strains of other *Neobacillus* species.

Compared genomes		ANI (%)	Genome A coverage for ANI (%)	Genome B Coverage for ANI (%)	dDDH (%)	95%CI (%)	POCP (%)
Genome A	Genome B
*N. nitrireducens* OS1-33^T^ [NZ_CP133262]	*N*.*drentensis*^T^ DSM 15600^T^ [GCF_055810755]	93.19	49.33	50.04	52.4	49.8–55.1	69
	*N. niacini* NBRC 15566^T^ [GCF_001591505]	74.48	30.55	25.71	20	17.8–22.4	11
	*N. bataviensis* LMG 21833^T^ [GCF_000307875]	77.28	32.29	31.64	21.1	18.9–23.6	18
	*N. novalis* NBRC 102450^T^ [GCF_001591805]	77.29	32.04	29.92	21.5	19.3–24	17
	*N. cucumis* DSM 101566^T^ [GCF_016908975]	76.03	31.63	27.19	21.1	18.9–23.5	13
	*N. rhizosphaerae* CIP 111895^T^ [GCF_937468385]	77.73	30.52	31.41	22.2	19.9–24.6	18
	N. soli DSM 15604^T^ [GCF_002335815]	78.77	34.52	31.91	23	20.7–25.5	21
	*N. vireti* DSM 15602^T^ [GCF_001026695]	77.23	30.31	29.51	21.7	19.4–24.1	16
	*N. mesonae* FJAT-13985^T^ [GCF_001636315]	74.41	29.01	25.78	21.1	18.9–23.5	8
	*N. ginsengisoli* DSM 27594^T^ [GCF_030813055]	76.11	31.44	30.01	21.1	18.9–23.6	13
	*N. pocheonensis* KCTC 13943^T^ [GCA_023702235]	76.57	30.06	24.73	22	19.7–24.4	12

The percentage of conserved proteins (POCP) between strain OS1-33^T^ and other *Neobacillus* species exceeded the genus boundary threshold of 50% only in comparison with *N. drentensis* (69%), whereas values for the other species ranged from 8 to 21%, well-below the 50% cutoff (Table 1; Qin et al., 2014). These results suggest that POCP may not be an appropriate criterion for defining the genus *Neobacillus*.

### Phenotypic traits

Strain OS1-33^T^ showed a close genomic relationship with *N. drentensis*, as presented in Table 2. However, several differences in phenotypic characteristics were observed between the two strains. Strain OS1-33^T^ formed white, smooth, and raised colonies on R2A agar, whereas *N. drentensis* DSM 15600^T^ formed white colonies with a rough surface and irregularly spreading morphology (Supplementary Figure S1).

**Table 2:** Morphological, physiological, and biochemical characteristics of *Neobacillus nitrireducens* OS1-33^T^ and its closely related *Neobacillus* species type strains.

Characteristics	1	2	3	4	5	6	7	8
Source	Apple orchard soil	Soil	Soil	Hay fields soil	Hay fields soil	Rhizosphere of cucumber	Soil	Soil
Gram stain	+	+	+	+	+	+	+	v
Cell size [Diameter (μm)/Length (μm)]	1.1–1.4/3.0–3.2	0.6–1.2/NR	0.9–1.4/3.0–5.6	0.7–1.2/NR	0.6–1.2/NR	NR/NR	0.6–1.2/NR	0.6–0.9/NR
Growth temperature (optimum; °C)	10–35 (30–35)	15–35 (35)	10–40	< 30– < 55 (30)	< 30– < 55 (30–40)	20–40 (20–30)	40–45 (30)	< 30– < 45 (30)
pH (optimum)	6–11 (7)	6–10 (6–8)	7–11.5 (7–8)	4–10 (7–8)	4–10 (7–9)	NR	4–9.5 (7–8)	5–10 (7–8)
Growth NaCl (optimum; %)	0–1	0–4	< 5	NR	NR	0.5–4 (1–2)	NR	NR
Catalase activity	+	+	(+)	NR	NR	+	NR	NR
Major quinone	MK−7	MK−7	NR	NR	NR	MK−7	NR	NR
Substrate utilization								
Glycerol	(+)	–	NR	(+)	v	NR	–	–
L-Arabinose	+	–	–	NR	NR	NR	NR	NR
Ribose	+	+	+	(+)	v	–	(+)	(+)
D-Xylose	+	–	–	NR	NR	NR	NR	NR
Galactose	+	(+)	NR	NR	NR	NR	NR	NR
Glucose	+	+	NR	NR	NR	NR	NR	NR
Fructose	+	+	NR	NR	NR	NR	NR	NR
Rhamnose	w	–	–	NR	NR	NR	NR	NR
Sorbitol	–	(+)	–	NR	NR	NR	NR	NR
Methyl-a-D-Glucosamine	+	(+)	NR	NR	NR	NR	NR	NR
N-Acethyl-Glucosamine	+	+	NR	NR	NR	NR	NR	NR
Amygdalin	+	–	NR	v	v	NR	–	–
Arbutin	+	–	+	NR	NR	NR	NR	NR
Esculin	+	–	+	NR	NR	NR	NR	NR
Salicin	+	+	+	(+)	–	–	–	–
Cellobiose	+	–	+	+	v	NR	–	–
Maltose	+	+	NR	NR	NR	NR	NR	NR
Lactose	+	+	+	+	–	–	–	–
Melibiose	+	+	NR	v	–	–	–	–
Sucrose	+	+	NR	v	–	–	v	+
Trehalose	(+)	(+)	NR	NR	NR	NR	NR	NR
Inulin	+	+	+	NR	NR	NR	NR	NR
Melezitose	+	+	+	–	+	–	–	–
Raffinose	(+)	(+)	–	+	–	–	–	–
Starch	(+)	+	NR	v	–	–	+	+
Gentiobiose	+	–	+	+	v	NR	–	–
D-Turanose	(+)	+	+	+	–	–	–	–
D-Lyxose	(+)	–	NR	NR	NR	NR	NR	NR
Gluconate	+	–	NR	NR	NR	NR	NR	NR
2-keto-Gluconate	–	(+)	NR	NR	NR	NR	NR	NR
5-keto-Gluconate	(+)	–	NR	NR	NR	NR	NR	NR
L-Fucose	–	–	NR	v	–	–	–	(+)

1, Neobacillus nitrireducens OS1-33^T^ (this study); 2, Neobacillus drentensis DSM 15600^T^ (Heyrman et al., 2004); 3, Neobacillus niacin NBRC 15566^T^ (Nagel and Andreesen, 1991); 4, Neobacillus bataviensis LMG 2183^T^ (Heyrman et al., 2004); 5, Neobacillus novalis NBRC 102450^T^ (Heyrman et al., 2004); 6, Neobacillus cucumis DSM 101566^T^ (Kämpfer et al., 2016); 7, Neobacillus soli R-16300^T^ (Heyrman et al., 2004); 8, Neobacillus vireti DSM 15602^T^ (Heyrman et al., 2004).

+, positive; –, negative; (+), weakly positive; v, results vary between strains; NR, not reported.

Although the growth temperature range (15°C−35°C), Gram staining, endospore formation, and catalase activity were identical for strain OS1-33^T^ and *N. drentensis* DSM 15600^T^, the pH range for growth differed slightly, being 6.0–11.0 for strain OS1-33^T^ and 6.0–10.0 for *N. drentensis* DSM 15600^T^ (Table 2).

Differences were also observed in carbon source utilization. Strain OS1-33^T^ was able to utilize 10 carbon sources that were not utilized by *N. drentensis*, including L-arabinose, D-xylose, galactose, methyl-α-D-glucoside, amygdalin, arbutin, esculin, cellobiose, gentiobiose, and gluconate. In contrast, starch and D-turanose were utilized only by *N. drentensis* (Table 2).

The predominant respiratory quinone of strain OS1-33^T^ was MK-7 (Table 1). The fatty acid C16:1 ω7c alcohol accounted for 5.62% of the total cellular fatty acids in strain OS1-33^T^, whereas it represented 9.78% in *N. drentensis* DSM 15600^T^, corresponding to an absolute difference of 4.16% (Table 3). In addition, several fatty acids were detected exclusively in specific strains. C15:1 iso F and C12:0 3OH were detected only in strain OS1-33^T^, whereas C17:1 iso ω10c and C17:0 cyclo were detected only in *N. drentensis* DSM 15600^T^ (Table 3).

**Table 3:** Cellular fatty acid contents (% of total fatty acids) of *Neobacillus* species type strain.

Cellular fatty acids	1	2	3	4	5	6	7	8
C_12:0_	0.41	0.32	NR	NR	NR	NR	NR	NR
C_12:0_ 3OH	0.42	NR	NR	NR	NR	NR	NR	NR
C_14:0_ iso	27.43	25.65	NR	6.9	5.3	4.3	3.3	3.1
C_14:0_	1.04	0.84	NR	1.5	3.0	2.6	< 1.0	1.6
C_15:1_ iso F	1.92	NR	NR	NR	NR	NR	NR	NR
C_15:0_ iso	12.07	10.43	NR	36.9	43.9	3.86	42.9	47.1
C_15:0_ anteiso	10.07	12.93	NR	20.5	31.0	3.32	33.5	1.9
C_15:0_	–	–	NR	NR	NR	NR	NR	NR
C_16:1_ w7c alcohol	5.62	9.78	NR	2.3	1.7	–	2.4	< 1.0
C_16:1_ iso H	1.43	0.51	NR	NR	NR	NR	NR	NR
C_16:0_ iso	18.97	16.99	NR	2.4	2.7	–	3.2	1.5
C_16:1_ wllc	3.98	6.03	NR	11.3	3.2	8.0	1.6	3.8
C_16:0_	7.05	6.71	NR	7.7	4.8	13.2	1.6	3.8
C_17:1_ iso w10c	NR	1.18	NR	1.7 ± 0.5	< 1.0	NR	4.1	< 1.0
C_17:0_ iso	1.48	1.44	NR	1.4	< 1.0	–	2.6	2.0
C_17:0_ anteiso	0.72	0.85	NR	1.1	1.9	–	1.5	2.7
C_17:1_ w9c	0.32	0.49	NR	NR	NR	NR	NR	NR
C_17:0_ cyclo	NR	0.23	NR	NR	NR	NR	NR	NR
C_17:0_	1.16	0.61	NR	NR	NR	NR	NR	NR
C_18:0_ iso	0.62	0.48	NR	NR	NR	NR	NR	NR
C_18:1_ w9c	0.47	1.02	NR	1.3	< 1.0	–	< 1.0	< 1.0
C_18:0_	1.27	1.41	NR	2.7	< 1.0	–	< 1.0	< 1.0
C_20:0_	0.34	0.32	NR	NR	NR	NR	NR	NR
Summed feature 3^*^	1.35	0.96	NR	NR	NR	NR	NR	NR
Summed feature 4^*^	0.30	0.62	NR	NR	NR	NR	NR	NR
Summed feature 8^*^	NR	0.21	NR	NR	NR	–	NR	NR
Summed feature 9^*^	1.60	NR	NR	NR	NR	NR	NR	NR

1, Neobacillus nitrireducens OS1-33^T^ (this study); 2, Neobacillus drentensis DSM 15600^T^ (this study); 3, Neobacillus niacin NBRC 15566^T^ (Nagel and Andreesen, 1991); 4, Neobacillus bataviensis LMG 2183^T^ (Heyrman et al., 2004); 5, Neobacillus novalis NBRC 102450^T^ (Heyrman et al., 2004); 6, Neobacillus cucumis DSM 101566^T^ (Kämpfer et al., 2016); 7, Neobacillus soli R-16300^T^ (Heyrman et al., 2004); 8, Neobacillus vireti DSM 15602^T^ (Heyrman et al., 2004).

–, not detected; NR, not reported.

These phenotypic differences, including carbon source utilization and cellular fatty acid profiles, support the conclusion that strain OS1-33^T^ and *N. drentensis* DSM 15600^T^ represent taxonomically distinct species.

### Nitrate (${\text{NO}}_{3}^{-}$) removal under various environmental conditions

**Figure 4:**
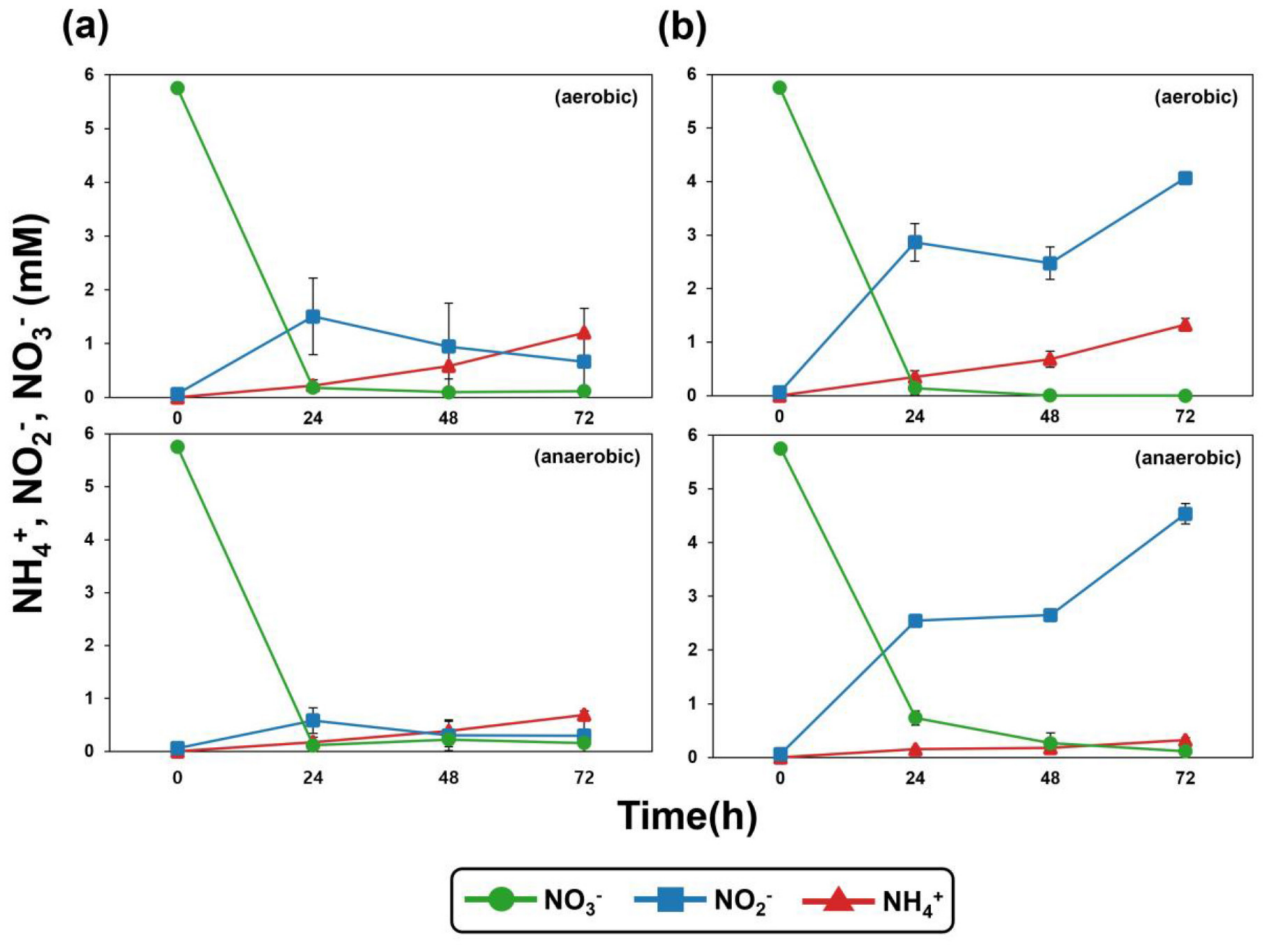
Changes in the concentrations of ${\text{NO}}_{3}^{-}$, ${\text{NO}}_{2}^{-}$, and ${\text{NH}}_{3}^{+}$ during aerobic and anaerobic incubation of **(a)** strain OS1-33^T^ and **(b)** *N. drentensis* DSM 15600^T^ in R2A broth supplemented with 5 mM nitrate and 10 mM acetate at 30°C with shaking at 100 rpm for 72 h.

Nitrate (${\text{NO}}_{3}^{-}$) removal was observed in the culture supernatants of both strain OS1-33^T^ and *N. drentensis* DSM 15600^T^ when cultivated in R2A broth under aerobic and anaerobic conditions (Figure 4). However, the key physiological difference between strain OS1-33^T^ and *N. drentensis* lies not in nitrate reduction itself, as both strains reduced nitrate under the tested conditions, but rather in the pattern of nitrite accumulation. *N. drentensis* did not efficiently reduce nitrite, resulting in its accumulation during nitrate reduction, whereas strain OS1-33^T^ efficiently reduced nitrite, thereby preventing its accumulation in the medium. Although both strains showed similar growth under aerobic and anaerobic conditions, differences in nitrite reduction performance were observed (Supplementary Table S3; Figure 4). This phenotypic difference was further supported by comparative genomic analysis. Strain OS1-33^T^ harbored an apparently intact *nirK* gene (RCG22_RS18465), whereas the corresponding loci in *N. drentensis* were annotated as *nirK* pseudogenes (AC0L1O_RS04870 and AC0L1O_RS13335). These pseudogene loci showed structural defects, including an internal stop codon and truncation at the N-terminus, which likely impair nitrite reductase function. This genomic difference is consistent with the observed phenotype, in which *N. drentensis* accumulated nitrite whereas strain OS1-33^T^ showed more efficient downstream nitrite reduction.

The genome of strain OS1-33^T^ further supports the denitrification ability of this strain; denitrification-related genes such as those for nitrate reduction (*narG*), nitrite reductase (*nirK*), nitric oxide reductase (*norB*), and nitrous oxide reductase gene (*nosZ*) were identified (Supplementary Table S4). In addition, a key gene for dissimilatory nitrate reduction to ammonium (DNRA), *nrfA*, encoding ammonia-forming nitrite reductase, was also identified on the genome of strain OS1-33^T^, indicating that this strain can perform both denitrification and DNRA, similar to some *Neobacillus* and other bacteria (Liu et al., 2021; Ahn et al., 2025). This also support the accumulation of ammonium in our incubation experiment (Figure 4).

Nitrate reduction is generally known to occur under anaerobic conditions, where (${\text{NO}}_{3}^{-}$) serves as the terminal electron acceptor (Pang and Wang, 2021). Nevertheless, several aerobic denitrifying microorganisms capable of utilizing both ${\text{NO}}_{3}^{-}$ and O_2_ simultaneously as electron acceptors under aerobic conditions have been reported (Robertson and Kuenen, 1984). Strain OS1-33^T^ maintained high nitrate removal activity up to 3.47 mg L^−1^ DO, whereas the activity decreased substantially at 7.72 mg L^−1^ (Table 4). These results indicate that strain OS1-33^T^ is capable of nitrate reduction under microaerobic conditions (DO ≤ 3.40 mg L^−1^).

**Table 4:** ${\text{NO}}_{3}^{-}$ removal (%) in R2A broth supplemented with 5 mM ${\text{NO}}_{3}^{-}$ and 10 mM acetate after incubation of strain OS1-33^T^ at 30°C for 48 h under different dissolved oxygen (DO) levels determined by shaking speed (rpm).

Shaking speed (rpm)	DO (mg/L)	Nitrate removal (%)
25	0.99 ± 0.05	96.77
50	1.7 ± 0.01	95.65
70	2.35 ± 0.05	95.16
80	3.47 ± 0.05	95.29
100	7.72 ± 0.01	35.75

Notably, strain OS1-33^T^ exhibited nitrate reduction activity in the presence of oxygen despite lacking *napA*, which encodes a periplasmic nitrate reductase commonly associated with nitrate reduction under aerobic conditions (Deng et al., 2020). This observation suggests that nitrate reduction in strain OS1-33^T^ may be mediated by alternative regulatory mechanisms, potentially involving *narG* (Supplementary Table S4), although further investigation is required to confirm its expression and activity under oxygenated conditions.

**Figure 5:**
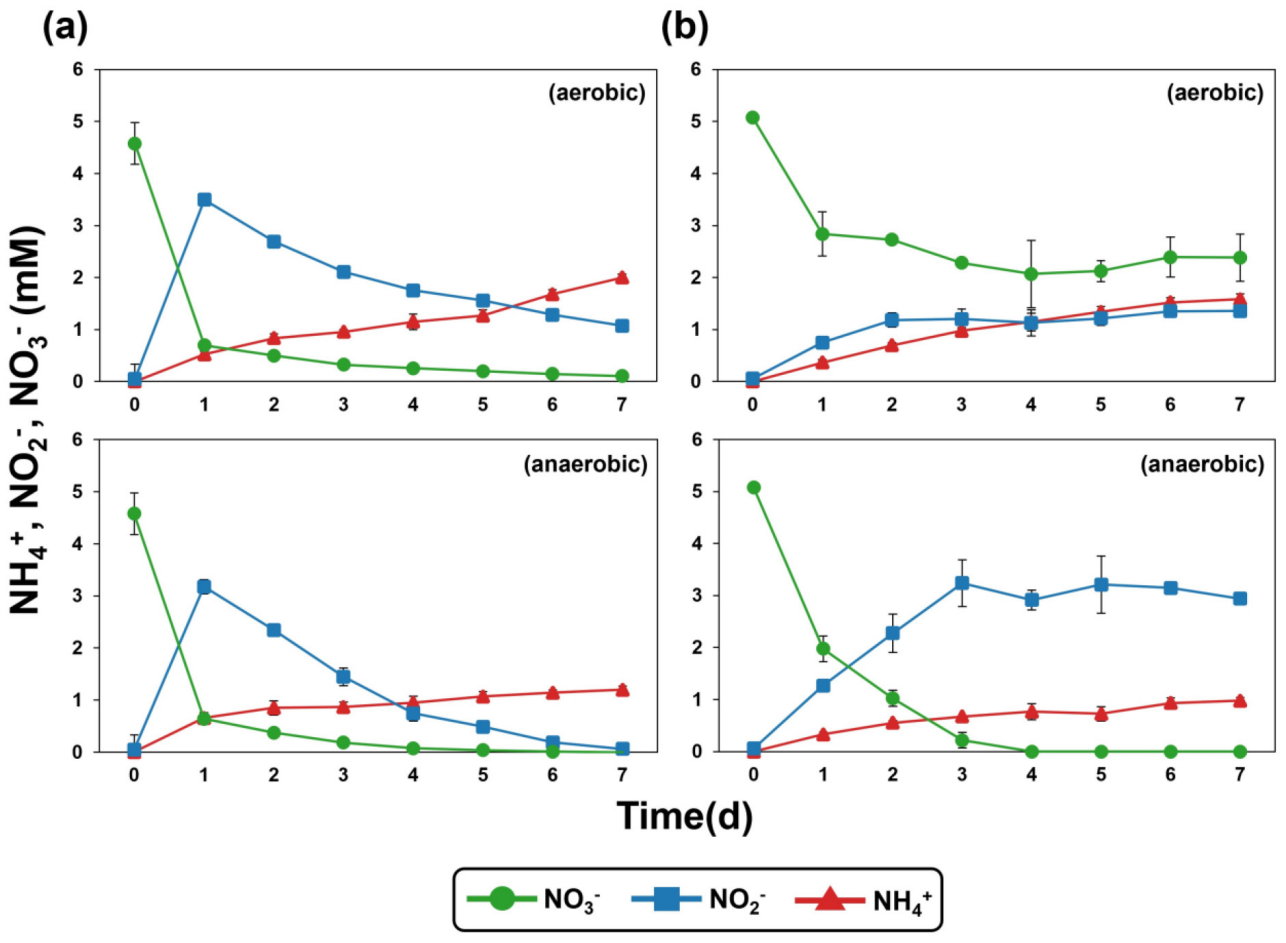
Changes in the concentrations of ${\text{NO}}_{3}^{-}$, ${\text{NO}}_{2}^{-}$, and ${\text{NH}}_{3}^{+}$ during aerobic and anaerobic incubation of **(a)** strain OS1-33^T^ and **(b)** *N. drentensis* DSM 15600^T^ with an initial OD_600_ of 2.2 in R2A broth supplemented with 5 mM nitrate and 10 mM acetate at 10°C with shaking at 100 rpm for 7 days.

Moreover, strain OS1-33^T^ maintained nitrate removal activity under low-temperature conditions in both the presence and absence of oxygen (Figure 5). The comparative experiment results between strain OS1-33T and *N. drentensis* were shown as an additional physiological property of strain OS1-33^T^ distinguishing it from *N. drentensis* in Figure 5. Under aerobic conditions at 10°C, *N. drentensis* reduced only half the amount of nitrate reduced by strain OS1-33^T^. Under anaerobic conditions at 10°C, *N. drentensis* completely reduced nitrate but did not efficiently reduce nitrite, in contrast to strain OS1-33^T^. Also, Genomic analysis revealed several genes potentially associated with low-temperature adaptation in strain OS1-33^T^, including multiple cold-shock protein genes, chaperone-related genes *(dnaK, dnaJ, groEL, groES*, and *clpB*), and membrane adaptation-related genes such as *desA* and several fatty acid biosynthesis genes (*fabD, fabG, fabI, fabF, fabL*, and *fabZ*; Supplementary Table S5). These features may contribute to the maintenance of protein stability and membrane fluidity under low-temperature conditions. Because biological nitrate removal is generally inhibited at low temperatures (Palacin-Lizarbe et al., 2018), this characteristic may be relevant to its potential application in nitrate-removing bioreactors operated under cold conditions (Christianson et al., 2012).

### Taxonomic conclusion

The 16S rRNA gene sequence similarity between strain OS1-33^T^ and *N. drentensis* IDA 1967^T^ (=DSM 15600^T^) was 99.93% with a query coverage of 98%, which exceeds the commonly accepted threshold for species delineation. However, the average nucleotide identity (ANI) value between the two strains was below 95%, which is widely regarded as the species boundary threshold.

Furthermore, strain OS1-33^T^ exhibited several phenotypic differences from *N. drentensis* DSM 15600^T^ and other members of the genus *Neobacillus*, including variations in cellular fatty acid composition and carbon source utilization. In addition, the nitrite reduction characteristic of strain OS1-33^T^ differed from those of *N. drentensis* DSM 15600^T^.

Taken together, the genomic, physiological, and chemotaxonomic characteristics support the conclusion that strain OS1-33^T^ represents a novel species within the genus *Neobacillus*. Therefore, strain OS1-33^T^ (=KCCM 43525^T^ = JCM 38007^T^) is proposed as the type strain of a novel species, for which the name *N. nitrireducens* sp. nov. is proposed.

### Description of *Neobacillus nitrireducens* sp. nov.

*Neobacillus nitrireducens* (ni.tri.re.du'cens. L. neut. n. *nitrum*, nitrate; L. pres. part. adj. *reducens*, reducing; N.L. part. adj. *nitrireducens*, nitrate-reducing).

**Figure 6:**
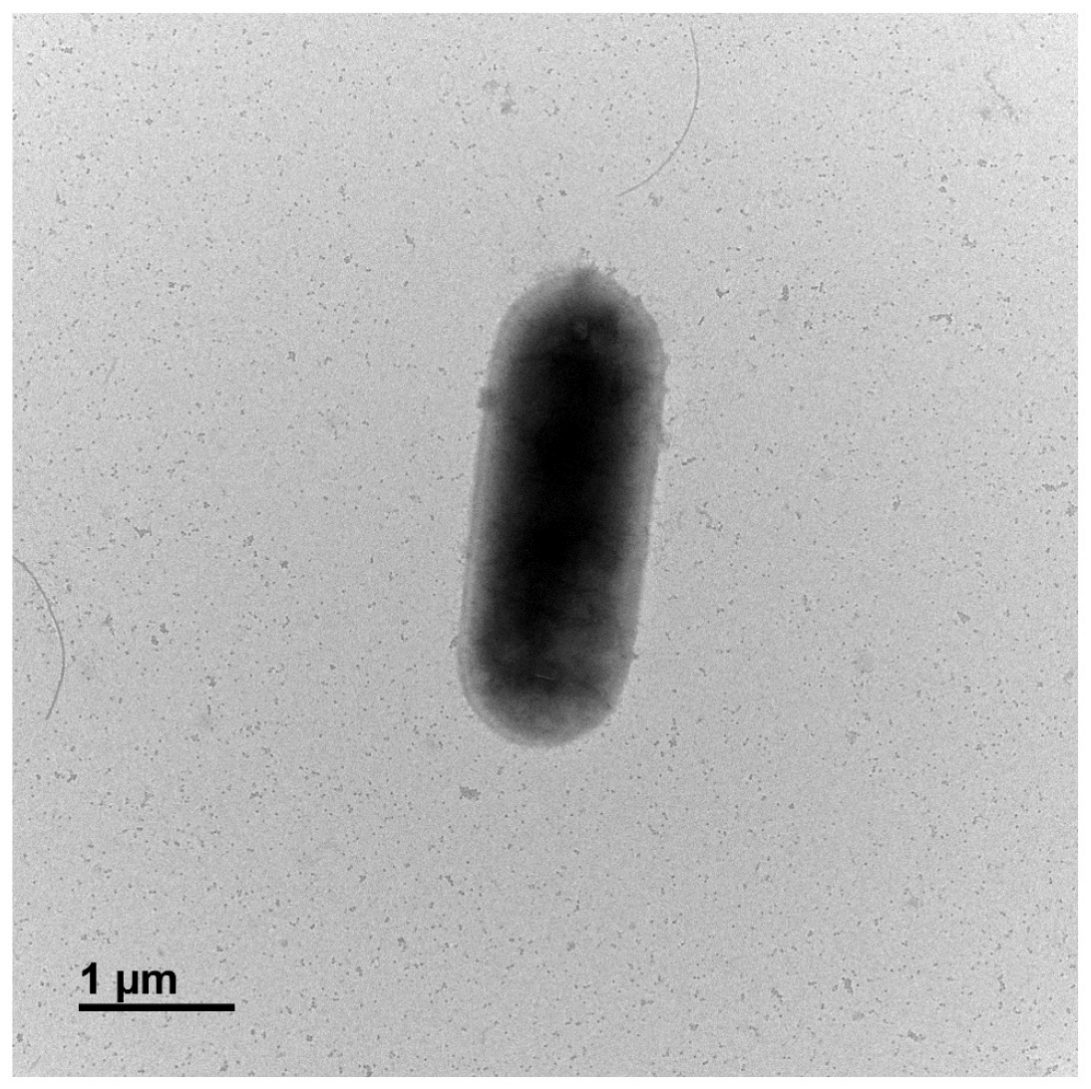
Transmission electron microscopic image of a cell of *Neobacillus nitrireducens* OS1-33^T^.

Cells are rod-shaped, measuring 1.1–1.38 μm in width and 3.0–3.2 μm in length (Figure 6). The strain is facultatively anaerobic and possesses flagella. Colonies on R2A agar are white, smooth, and raised. Growth occurs at 10°C−35°C (optimum 30°C−35°C) and at pH 6–11 (optimum pH 7). Growth occurs in the presence of 0%−1% (w/v) NaCl on R2A agar. The strain exhibits nitrate-reducing activity, including under aerobic and low-temperature conditions (e.g., 10°C). Utilizable carbon sources include L-arabinose, ribose, D-xylose, galactose, glucose, fructose, methyl-α-D-glucoside, N-acetylglucosamine, amygdalin, arbutin, esculin, salicin, cellobiose, maltose, lactose, sucrose, inulin, melezitose, melibiose, gentiobiose, and gluconate. The major cellular fatty acids are iso-C14:0 and iso-C16:0. The predominant respiratory quinone is MK-7. The genomic DNA G+C content of the type strain is 38.92%. The species can be differentiated from its closest relative, *N. drentensis*, by its smooth and raised colony morphology, ability to grow at pH 11, distinct carbon source utilization profile, differences in cellular fatty acid composition, and efficient nitrite reduction during nitrate transformation under the tested conditions. The strain also shows nitrate-reducing activity under the tested conditions, including under aerobic conditions and at low temperature (10°C). The type strain, OS1-33^T^ (=KCCM 43525^T^ = JCM 38007^T^), was isolated from soil of an apple orchard in Iksan, Republic of Korea.

The 16S rRNA gene and whole-genome sequences of strain OS1-33^T^ have been deposited in GenBank under the accession numbers PV185345 and NZ_CP133262, respectively.

## Data Availability

The datasets presented in this study can be found in online repositories. The names of the repository/repositories and accession number(s) can be found in the article/Supplementary material.
