# Supplementary material for: Neobacillus nitrireducens sp. nov., a soil bacterium performing nitrate reduction under low-temperature and microaerobic conditions
Source: Front Microbiol. 2026 May 11;17:1842410. doi: 10.3389/fmicb.2026.1842410 (PMC13199352; doi:10.3389/fmicb.2026.1842410)
Supplement: Supplementary file 1 [file Table_1.docx]

Supplementary Information

***Neobacillus nitrireducens* sp. nov., a soil bacterium performing nitrate reduction under low-temperature and microaerobic conditions**

Jinwoo Ahn^1^, Satoshi Ishii^2,3^, Tatsuya Unno^4*^, and Jeonghwan Jang^1*^

^1^Division of Biotechnology and Advanced Institute of Environment and Bioscience, Jeonbuk National University, Iksan, Jeonbuk 54596, Republic of Korea

^2^BioTechnology Institute, University of Minnesota, St. Paul, MN 55108, USA

^3^Department of Soil, Water, and Climate, University of Minnesota, St. Paul, MN 55108, USA

^4^Department of Biological Sciences and Biotechnology, Chungbuk National University, Seowon-gu, Cheongju 28644, Republic of Korea

^*^ Corresponding author: Dr. Jeonghwan Jang, Division of Biotechnology, Jeonbuk National University, 79 Gobong-ro, Iksan, Jeonbuk 54596, Republic of Korea. Email: [jangj@jbnu.ac.kr](mailto:jangj@jbnu.ac.kr)

^*^ Co-corresponding author: Dr. Tatsuya Unno, Department of Biological Sciences and Biotechnology, Chungbuk National University, Seowon-gu, Cheongju 28644, Republic of Korea. Email: [tatsu@cbnu.ac.kr](mailto:tatsu@cbnu.ac.kr)

Table S1. Genome sequencing summary for *Neobacillus nitrireducens* OS1-33^T^ and *Neobacillus drentensis* DSM 15600^T^.

| Attribute | OS1-33^T^ | DSM 15600^T^ |
| --- | --- | --- |
| Total sequence reads | 5,153,056 bp | 5,293,193 bp |
| Number of contigs | 1 | 1 |
| Genome coverage | 222× | 137× |
| Number of CDSs | 4,896 | 5,029 |
| Number of tRNAs | 126 | 127 |
| Number of rRNA genes (5S, 16S, 23S) | 17, 17, 17 | 17, 17, 17 |
| GenBank accession no. | NZ_CP133262 | CM147946 |

Table S2. GC content ratio between strain OS1-33^T^ and type strains of other *Neobacillus* species: 1, *Neobacillus nitrireducens* OS1-33^T^ (this study); 2, *Neobacillus drentensis* DSM 15600^T^ (this study); 3*, Neobacillus niacin* NBRC 15566^T^; 4, *Neobacillus bataviensis* LMG 21833^T^; 5, *Neobacillus novalis* NBRC 102450^T^; 6, *Neobacillus cucumis* DSM 101566^T^. ; 7, *Neobacillus rhizosphaerae* CIP 111895^T^; 8, *Neobacillus soli* DSM 15604^T^; 9, *Neobacillus vireti* DSM 15602^T^ ; 10, *Neobacillus mesonae* FJAT-13985^T^; 11, *Neobacillus ginsengisoli* DSM 27594^T^; 12, *Neobacillus pocheonensis* KCTC 13943^T^.

| strain | GenBank accession no. | | GC content (%) | |
| --- | --- | --- | --- | --- |
| 1 | NZ_CP133262 | 38.92 | |  |
| 2 | CM147946 | 38.93 | |  |
| 3 | GCF_001591505 | 38.16 | |  |
| 4 | GCF_000307875 | 39.61 | |  |
| 5 | GCF_001591805 | 39.86 | |  |
| 6 | GCF_016908975 | 38.32 | |  |
| 7 | GCF_937468385 | 38.32 | |  |
| 8 | GCF_002335815 | 39.71 | |  |
| 9 | GCF_001026695 | 39.78 | |  |
| 10 | GCF_001636315 | 40.31 | |  |
| 11 | GCF_030813055 | 38.10 | |  |
| 12 | GCA_023702235 | 37.87 | |  |

Table S3. Growth (OD_600_) of OS1-33^T^ and DSM 15600^T^ under aerobic and anaerobic conditions at 30°C up to 72 h. Values represent the mean of triplicate experiments, and error bars indicate the standard deviation (SD). This table corresponds to the data presented in Fig. 2.

| strain | condition | 24 h (OD_600_) | 48 h (OD_600_) | 72 h (OD_600_) |
| --- | --- | --- | --- | --- |
| OS1-33^T^ | aerobic | 0.684±0.158 | 0.759±0.146 | 0.786±0.128 |
|  | anaerobic | 0.575±0.064 | 0.461±0.005 | 0.442±0.010 |
| DSM15600^T^ | aerobic | 0.689±0.050 | 0.878±0.028 | 0.871±0.054 |
|  | anaerobic | 0.217±0.007 | 0.266±0.036 | 0.259±0.041 |

Table S4 . Genes involved in denitrification and DNRA identified from the genome of *Neobacillus nitrireducens* OS1-33^T^

| gene | locus_tag | product |
| --- | --- | --- |
| *narG* | RCG22_RS02395 | nitrate reductase  subunit alpha |
| *nirK* | RCG22_RS18465 | multicopper oxidase domain-containing protein |
| *norB* | RCG22_RS11585 | nitric-oxide reductase large subunit |
| *nosZ* | RCG22_RS16135 | Sec-dependent nitrous-oxide reductase |
|  | RCG22_RS21460 |  |
| *nrfA* | RCG22_RS20820 | ammonia-forming cytochrome c nitrite reductase subunit c552 |

Table S5. Genes potentially associated with low-temperature adaptation. Specifically, the genome contains multiple cold-shock protein (CSP) genes that, based on BLASTP analysis, show high similarity to the *cspA* family (RCG22_RS00270 and RCG22_RS04740) and *cspD* family (RCG22_RS05220, RCG22_RS05460, RCG22_RS24100, and RCG22_RS24595).

| Cold Adaptation | Gene | Locus_tag | Product |
| --- | --- | --- | --- |
| cold-shock response | *cspD* | RCG22_RS24100 | cold-shock protein *CspD* |
|  | Cold-Shock Protein Gene | RCG22_RS00270  RCG22_RS04740  RCG22_RS05220  RCG22_RS05460  RCG22_RS24595  RCG22_RS24600 | a cold-shock protein |
| chaperone system | *dnaK* | RCG22_RS22330 | molecular chaperone *DnaK* |
|  | *naJ* | RCG22_RS22335 | as molecular chaperone *DnaJ* |
|  | *groEL* | RCG22_RS14335 | chaperonin *GroEL* |
|  | *groES* | RCG22_RS14340 | co-chaperone *GroES* |
|  | *clpB*: | RCG22_RS09490 | ATP-dependent chaperone *ClpB* |
| membrane adaptation | *desA* | RCG22_RS13125 | Delta(5) desaturase *DesA* |
|  | *fabG* | RCG22_RS03550 | 3-oxoacyl-ACP reductase *FabG* |
|  | *fabG* | RCG22_RS07815 | 3-oxoacyl-[acyl-carrier-protein] reductase |
|  | *fabD* | RCG22_RS07820 | ACP S-malonyltransferase |
|  | *fabI* | RCG22_RS09305 | enoyl-ACP reductase *FabI* |
|  | *abF* | RCG22_RS09455 | beta-ketoacyl-ACP synthase II |
|  | *fabL* | RCG22_RS10670 | enoyl-[acyl-carrier-protein] reductase *FabL* |
|  | *fabZ* | RCG22_RS16675 | 3-hydroxyacyl-ACP dehydratase *FabZ* |


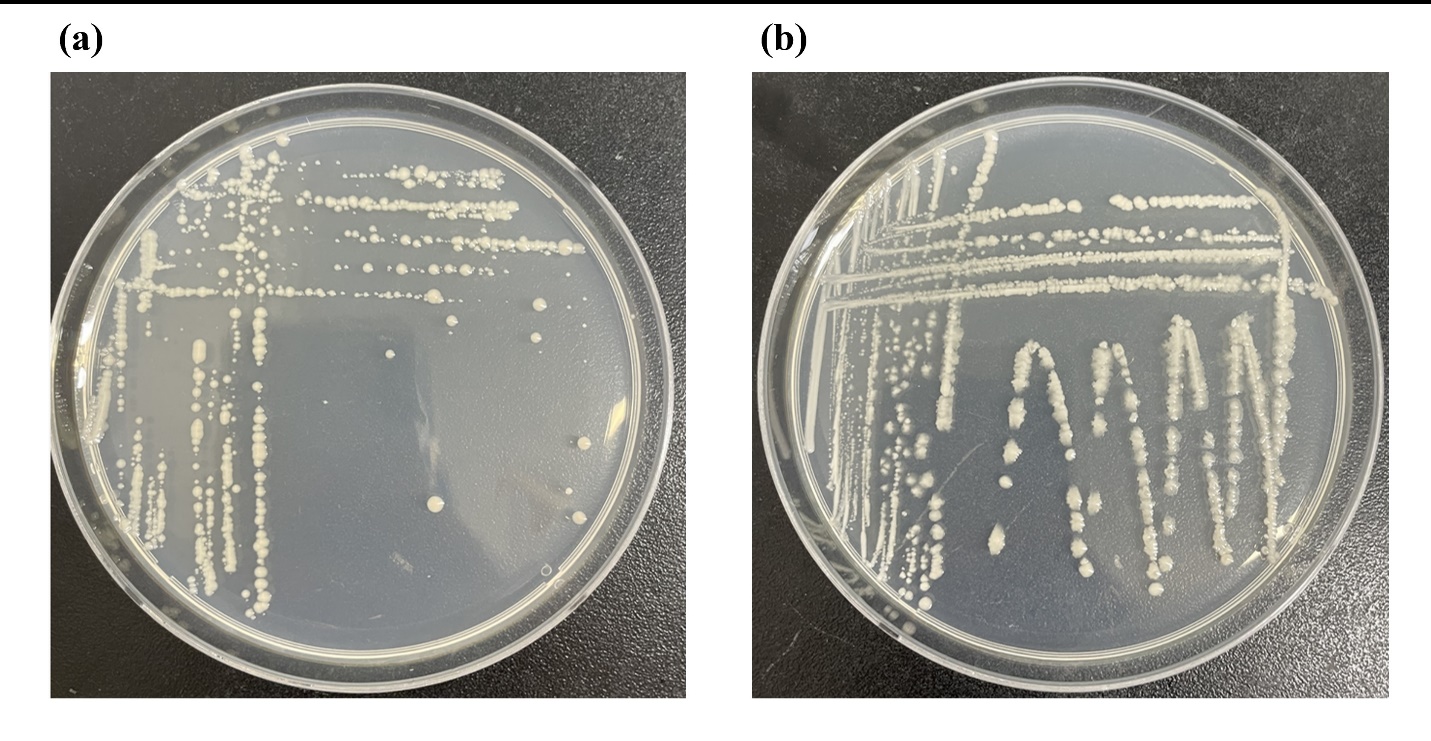
Fig. S1. Colonies of (a) OS1-33ᵀ and (b) *N. drentensis* DSM15600ᵀ on R2A agar after incubation at 30°C for 2 days.
